# Human Ace D/I Polymorphism Could Affect the Clinicobiological Course of COVID-19

**DOI:** 10.1155/2021/5509280

**Published:** 2021-09-15

**Authors:** Elifcan Aladag, Zahit Tas, Bilgesu Safak Ozdemir, Tayfun Hilmi Akbaba, Meltem Gulsun Akpınar, Hakan Goker, Tugce Unalan-Altintop, Ahmet Cagkan Inkaya, Alpaslan Alp, Gokhan Metan, Ibrahim Celalettin Haznedaroglu, Banu Balci-Peynircioglu, Nilgun Sayinalp

**Affiliations:** ^1^Department of Hematology, Hacettepe University Faculty of Medicine, Ankara, Turkey; ^2^Department of Infectious Diseases, Hacettepe University Faculty of Medicine, Ankara, Turkey; ^3^Department of Medical Biology and Genetics, Hacettepe University Faculty of Medicine, Ankara, Turkey; ^4^Department of Radiology, Hacettepe University Faculty of Medicine, Ankara, Turkey; ^5^Department of Microbiology and Clinical Microbiology, Hacettepe University Faculty of Medicine, Ankara, Turkey

## Abstract

**Introduction:**

The coronavirus disease 2019 (COVID-19), that is caused by severe acute respiratory syndrome corona virus 2 (SARS-CoV-2), has spread rapidly worldwide since December 2019. The SARS-CoV-2 virus has a great affinity for the angiotensin-converting enzyme-2 (ACE-2) receptor, which is an essential element of the renin-angiotensin system (RAS). This study is aimed at assessing the impact of the angiotensin-converting enzyme (ACE) gene insertion (I)/deletion (D) polymorphisms, on the susceptibility and clinical outcomes of the COVID-19 immunoinflammatory syndrome. *Patients and Methods*. A total of 112 patients diagnosed with COVID-19 between 1 and 15 May 2020 were enrolled in the study. ACE gene allele frequencies were compared to the previously reported Turkish population comprised of 300 people.

**Results:**

The most common genotype in the patients and control group was DI with 53% and II with 42%, respectively. The difference in the presence of the D allele between the patient and control groups was statistically significant (67% vs. 42%, respectively, *p* < 0.0001). Severe pneumonia was observed more in patients with DI allele (31%) than DD (8%) and II (0%) (*p* = 0.021). The mortality rate, time to defervescence, and the hospitalization duration were not different between the genotype groups.

**Conclusion:**

Genotype DI of ACE I/D polymorphism is associated with the infectious rate particularly severe pneumonia in this study conducted in the Turkish population. Therefore, ACE D/I polymorphism could affect the clinical course of COVID-19.

## 1. Introduction

Severe acute respiratory syndrome coronavirus 2 (SARS-CoV-2) is a novel coronavirus subtype from the Sarbecovirus virus family, causing the currently expanding coronavirus disease 2019 (COVID-19) pandemic [1]. As previously demonstrated with severe acute respiratory syndrome coronavirus, this virus also has a high-affinity potential for the human angiotensin-converting enzyme-2 (ACE-2) [2]. The virus adheres to the nasopharyngeal and alveolar surfaces expressing ACE-2 through the spike proteins located at the viral envelope [3]. The tissue-based renin-angiotensin system (RAS) plays an essential role in the pathobiology of COVID-19 [4, 5]. While the angiotensin-converting enzyme-1 (ACE-1) enzyme mediates the formation of angiotensin-2, ACE-2 converts angiotensin-2 to angiotensin-1 by hydrolyzing. That balance is crucial for the genesis of the syndrome, since the imbalance in favor of the angiotensin-2 is implicated in the mechanism exacerbating COVID-19 such as vasoconstriction, fibrosis, inflammation, and thrombosis [6]. Hence, acquired and/or congenital alterations regulating the expression or function of RAS impacts the development and clinical course of COVID-19 [7].

Angiotensin-converting enzyme (ACE) insertion (I)/deletion (D) polymorphisms are one of the most frequently defined human polymorphisms. (D) and (I) polymorphisms in the ACE gene in populations may result in differences in ACE levels. For instance, the ACE D allele causes an increase in ACE-1 level and a decrease in ACE-2 level, causing an increased level of angiotensin-2 and progression of pulmonary edema, through increased microvascular permeability. That phenomenon further worsens the clinical course and prognosis in the diseases such as acute respiratory distress syndrome (ARDS) [8]. Indeed, the protective effect of ACE-2 in acute pulmonary syndrome has been shown in experimental studies, and angiotensin-2 stimulation could be an important mechanism that could be used for the management of acute lung injuries. Likewise, the demonstration of 30-day mortality in ARDS patients with ACE DD genotype, opposed to ID or II genotype, may be conceived as a clinical implication of this model [9, 10].

The aim of this study is to assess the impact of ACE gene polymorphism on the susceptibility and clinical outcomes of COVID-19. Elucidation of the impact of RAS elements, including ACE, could be helpful for better understanding the pathobiology of COVID-19 as well as the clinical management of patients infected with SARS-CoV-2 [11].

## 2. Patients and Methods

### 2.1. Study Population

A total of 112 patients with PCR positive-COVID-19 who were diagnosed between 1 and 15 May 2020 were enrolled in this crosssectional single-center study. All patients are over 18 years of age, and each had a positive SARS-CoV-2 reverse transcription-polymerase chain reaction (RT-PCR) test result taken from the upper respiratory swab. Treatment and discharge decisions were held by attending physicians according to the current national guidelines prepared by a Scientific Advisory Committee of the Turkish Ministry of Health. Institutional review board approval was granted from the Hacettepe University Ethical Committee for noninterventional studies (GO 20/618).

Patients with pneumonia were classified as severe or nonsevere. Severe pneumonia was defined as the presence of fever (38 degrees Celsius or above) or suspected respiratory infection, plus one of the following: respiratory rate > 30 breaths/min; severe respiratory distress; or oxygen saturation measured by pulse oximetry (SpO_2_) ≤ 93% on room air [12]. The demographics and medical information of the subjects were obtained according to patients' declaration. Vital signs (temperature, pulse, respiration rate, pulse oxygen saturation, and blood pressure) were recorded daily. Laboratory tests for complete blood count, alanine aminotransferase (ALT), aspartate aminotransferase (AST), lactate dehydrogenase (LDH), blood urea nitrogen (BUN), creatinine, C-reactive protein (CRP), neutrophil to lymphocyte ratio (NLR), D-dimer, ferritin, and creatine kinase (CK) levels were recorded. As a control group, 300 patients from a large-scale study conducted in the Turkish population were accepted as reference [13].

### 2.2. Deoxyribonucleic Acid Isolation

Extraction of genomic deoxyribonucleic acid (DNA) from the peripheral blood of COVID-19 patients was performed according to a revised version of the previously published salting-out protocol by the Department of Clinical Microbiology, Hacettepe University [14]. Peripheral blood was collected in 10 milliliter (mL) ethylenediaminetetraacetic acid tubes, transferred to 50 mL tubes, and completed to 45 mL with autoclaved water stored in +4°C for physical fragmentation, then shaken vigorously for homogenization. After that, samples were centrifuged at 3500 rpm for 15 minutes at 20°C. After centrifugation, the supernatant was discarded, and 3 mL nucleic lysis buffer, 150 microliters (*μ*L) proteinase K, and 200 *μ*L SDS were added to the pellet and vortexed until homogenized, then incubated overnight (O/N) at 37°C, after incubation, 3 mL 10 M ammonium acetate was added to tubes and incubated for another 10 minutes at room temperature. After centrifugation at 4000g for 20 minutes, the supernatant was transferred to another 50 mL tube, and ice-cold 100% ethyl alcohol was added. DNA strands formed, collected, and resuspended in 350 *μ*L TE buffer and incubated O/N at 37°C. The purity and concentration of extracted DNA samples of patients were measured by using Nanodrop 200 Spectrometer (Thermo Scientific, USA). DNA samples with at least 50 nanogram/*μ*L and A260/280 ratio of ~1.8 were selected for further analysis.

### 2.3. Polymerase Chain Reaction

**Figure 1:**
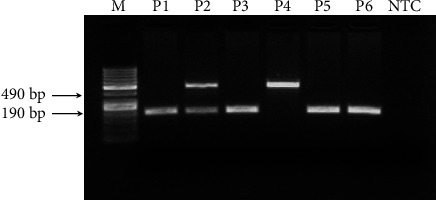
PCR products on agarose gel electrophoresis. I allele: 490 bp, D allele:190 bp. M: 100 bp DNA marker; P1, P3, P5, P6: DD polymorphism; P2: DI polymorphism; P4: II polymorphism; NTC: nontemplate control.

**Figure 2:**
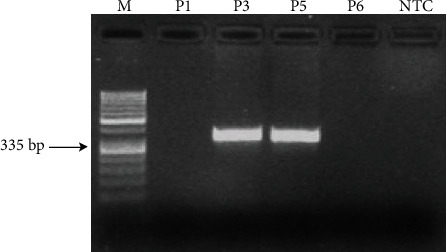
PCR products on agarose gel electrophoresis with use of insertion-specific primers. I: 335 bp. M: 100 bp DNA marker; P1, P6: DD polymorphism; P3, P5: DI polymorphism. NTC: nontemplate control.

Two different PCR reactions were performed with two different primer sets to define deletion/insertion polymorphisms of the ACE gene in COVID-19 patients [15]. Both PCR reactions were conducted in a 25 *μ*L reaction volume in a SimpliAmp™ Thermal Cycler (Applied Biosystems) using Taq DNA polymerase (Thermo Scientific) (Figure 1). Primer sets of both PCR reactions were given in Table 1. The optimized PCR cycling condition for the first PCR reaction was as follows: 94°C for 2 min, followed by 30 cycles of 94°C for 30 s (denaturation), 53°C for 1 min (annealing), and 72°C for 1 min (extension), followed by 72°C 3 min final step. Samples that only have insertion-specific PCR products are defined as II genotypes, and samples with both insertion and deletion-specific products are defined as DI genotypes. However, insertion-specific PCR products (490 bp) can be suppressed in the first PCR reaction in some cases because amplification of shorter DNA segments can be selected in the reaction. The second PCR reaction was performed for samples with DD genotype according to the first PCR results [15]. This PCR was conducted with insertion-specific primers (Table 1). PCR cycling condition for insertion-specific PCR was as follows: 94°C for 2 min, followed by 30 cycles of 94°C for 30 s (denaturation), 69°C for 1 minute (annealing), and 72°C for 1 minute (extension), followed by 72°C for 3 minutes. As a result of the second PCR, amplification (355 bp) detected samples were defined as DI genotype, whereas samples without amplification are defined as DD genotype (Figure 2). After the first PCR, II and DI genotypes were determined, second PCR samples that result in amplification were defined as DI genotypes additional to ones in the first PCR and ones that do not give amplification detected as DD genotypes. As a result of these two PCR reactions, polymorphic allele frequencies of the ACE gene were determined in COVID-19 patients.

### 2.4. Statistical Analysis

IBM SPSS Statistics for MacOS, version 25.0 (IBM Corp., Armonk, N.Y., USA), were used in statistical analyses. The variables were investigated using visual (histograms, probability plots) and analytical methods (Kolmogorov-Simirnov/Shapiro-Wilk's test) to determine whether they are normally distributed. Descriptive analyses were presented using medians and interquartile range for the nonnormally distributed variables. Mean ± standard deviation, median [minimum-maximum], and frequency were used as descriptive statistics. Chi-square test or Fisher exact test was implemented to compare categorical variables, and Student's *t*-test, Mann-Whitney *U* test, or Kruskal-Wallis test was used to compare continuous variables, where appropriate. A *p* value of less than 0.05 was considered to show statistically significant results.

### 2.5. Ethical Considerations

The research protocol was reviewed and approved by the Hacettepe University Ethical Committee for noninterventional studies (approval number: GO 20/618). All patients gave informed consent for collecting personal data for research purposes. All ethical considerations were strictly followed in accordance with the Declaration of Helsinki.

## 3. Results

### 3.1. Detection of D/I Polymorphisms in COVID-19 Patients with Polymerase Chain Reaction

Figure 1 presents the results of the first PCR. II genotype corresponds to samples that resulted in a 490 bp product and those resulted in two bands at 190 bp and 490 bp detected as heterozygous genotype DI. For the samples that only 190 bp band detected, a second insertion-specific PCR was applied. Figure 2 shows the second PCR for those samples that resulted in 190 bp in the first one. Samples with a band around 335 bp after second PCR genotyped as DI, while those do not result in a band genotyped as DD.

### 3.2. Analysis of D/I Allele Frequencies in Turkish COVID-19 Patients

The frequencies of the DD, DI, and II genotypes of ACE in the control group were 26%, 32%, and 42%, respectively. The D allele frequency was 42%, and the I allele frequency was 58%. In contrast, the frequencies of DD, DI, and II genotypes of ACE in the COVID-19 patients were 40%, 53%, and 7%, respectively; the D allele frequency was 67%, and the I allele frequency was 33%.  Table 2 depicts the distribution of ACE I/D polymorphisms in the COVID-19 patients and controls. The difference in the presence of the D allele between the patient and control groups was statistically significant (67% vs. 42%, respectively, *p* < 0.0001).

### 3.3. Genotype-Phenotype Correlation in COVID-19 Patients

Table 3 depicts the baseline characteristics of the studied COVID-19 patients. There was no difference in terms of these parameters among different genotypes. The most common comorbidity observed in the patients with DD and DI genotype was hypertension. Four of the patients had malignancies: lung adenocarcinoma, prostate carcinoma, T cell lymphoma, and breast cancer.

**Figure 3:**
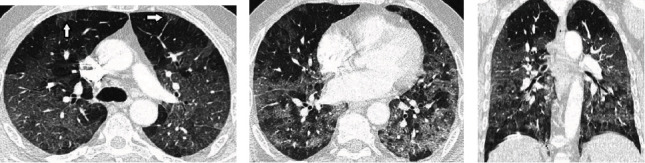
Radiological evaluation of severe COVID-19 pneumonia in a patient with DI genotype. A 60-year-old man with severe headache admitted to the emergency and had a positive RT-PCR test result for SARS-CoV-2. Thin section thorax CT images ((a, b) axial images, (c) coronal reformat) show bilateral diffuse ground-glass opacities with superimposed interlobular septal thickening (arrows) representing severe COVID-19 pneumonia.

**Figure 4:**
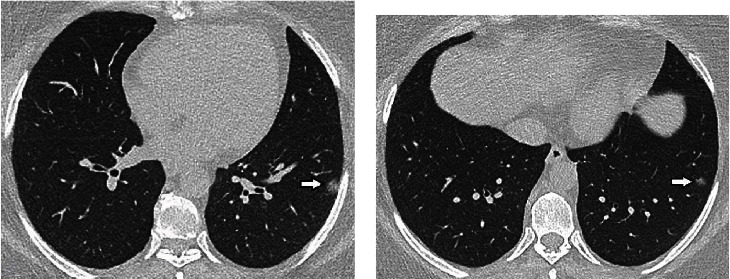
Radiological evaluation of nonsevere COVID-19 pneumonia in a patient with II genotype. A 47-year-old woman with a complaining of muscle ache had a positive RT-PCR test result for SARS-CoV-2. Axial nonenhanced CT scan of the chest ((a, b) axial images) shows areas of peripheral rounded ground-glass opacities in the left lower lobe (arrows) representing mild COVID-19 pneumonia.

All patients with the II allele had pneumonia; however, none of them required oxygen supplementation. While patients with the DI allele (31.1%) had significantly more severe pneumonia (Figure 3), none of the patients with II allele had severe pneumonia (Figure 4), and 8% of the patients with DD allele had severe pneumonia (*p* = 0.021). A high-grade fever was present in 51.1%, 45.8%, and 37.5% of those with DD, DI, and II alleles, respectively. However, the difference between the groups was not statistically significant (*p* = 0.730) (Table 4).

The median duration of fever reduction in all genotypes was three days (*p* = 0.09). The mortality in the DI allele group (6.8%) was higher than in the DD (2.2%) and the II (0%) groups but not significant (Table 5). Two of the five patients who died during the study had been receiving active cancer treatment.

## 4. Discussion

Gene polymorphisms of ACE has been the subject of debate since the beginning of the pandemic. The variants of ACE-2 and the receptor of SARS-CoV-2, caused by gene polymorphism, are thought to result in differences in disease susceptibility and disease severity [7]. Previous observations confirmed that the incidence of pneumonia was higher in patients carrying D allele in SARS-CoV-1 infection [16]. This has led the researchers to further investigate the relationship between COVID-19 and ACE gene polymorphisms. ACE polymorphism with regard to the COVID-19 outcome seems to be affected by the presence of ACE1 D/I polymorphism [17–19]. The distribution of ACE gene polymorphisms and their impact on the outcomes of 112 COVID-19 patients are studied in this present research. Our findings indicate that the prevalence of DI genotype and D-allele carriage is common among COVID-19 patients compared to the Turkish population. When the disease severity was investigated depending on ACE gene polymorphism genotypes, the patients with DI genotype had a more severe clinical course and higher mortality than those with DD and II genotypes.

Racial differences of ACE gene polymorphisms are extensively studied. In a previous study, the frequency of the D alleles is higher in African American populations (89%) than that in Indian and Caucasian (69%) [20]. Similarly, in some European countries, especially in the Mediterranean basin such as Italy, France, and Spain, the frequency of the D alleles can be present up to 87%. On the other hand, it has been shown that the frequency of II allele in Asian countries is higher than that in Europe [21, 22]. Therefore, the devastating effects of COVID-19 among the African American population and Europeans, where D allele is more common, are attributed to this allelic distribution. In a polymorphism study conducted in Asian countries, there was a significant correlation between D allele and mortality (*r* = 0.602; *p* = 0.002), but there was no significant correlation between recovery rate and alleles (*r* = −0.200; *p* = 0.350) consistent with our findings [23].

There are different hypotheses to explain why mortality is higher in patients carrying the D allele. The most discussed and popular one is that the negative correlation between D allele and ACE-2 levels and eventually exacerbated lung injury due to inflammatory effects of angiotensin-2 which increases as ACE-2 level decreases [24]. Parallel to this hypothesis, it has been shown that the frequency of ARDS and associated mortality increase with the presence of the D allele in some studies [25, 26]. The present study results are consistent with the previous studies and showed that while patients with the II genotype did not develop severe pneumonia, those with the DI genotype experienced statistically significant severe pneumonia and an increase in mortality, despite the lack of statistical significance. In addition, the increased mortality in patients carrying the D alleles can also be attributed to the increased incidence of some comorbidities such as coronary artery disease, hypertension, diabetes mellitus, and dyslipidemia in the presence of the D allele [27, 28]. Since we could not demonstrate any statistical difference in comorbidity among different genotype groups, it is difficult to speculate that different genotypes affected outcomes through comorbidities. However, large-scale studies are needed to evaluate such a theory.

The studies assessing the relation between ACE gene polymorphism and infectious risk is scarce. In our study, there was a positive correlation between the number of cases and the presence of the D allele (*r* = 0.502; *p* = 0.008). On the contrary, a study conducted at 25 European countries in the beginning of the pandemic, a negative correlation between the D allele frequency and the number of cases (*r* = −0.510; *p* = 0.01), and a positive correlation between the D allele frequency and mortality (*r* = 0.370; *p* = 0.001) were found [29]. In this study, the theory that the frequency of the D allele decreases infectivity is attributed to the decrease in ACE-2 level, which acts as a receptor for SARS-CoV-2. Different sensitivities to SARS-CoV-2 infection may arise from ACE polymorphism variability through affecting ACE-2 stability and virus binding [30]. Moreover, the entry of the SARS-CoV virus into the cell is due to the possible upregulation of the ANPEP gene, which is involved in the initial phase with ACE-2 and is also a member of the RAS family [11]. ACE2 receptor is expressed on the surface of hematopoietic stem cells within the context of local bone marrow RAS, representing a target for SARS-CoV-2 attack on bone marrow hematopoiesis [31, 32]. Previously, interrelationships between hematopoietic RAS, lymphoid malignancies, and ACE polymorphisms had been demonstrated [33, 34]. The latest hematological research disclosed the importance of ACE-hematopoietic stem cell dynamics during the progression of COVID-19 [2, 31, 35]. Thus, the alterations of ACE molecules in distinct tissue/organ microenvironments could be associated with the heterogeneous clinical presentations of the COVID-19 syndrome.

In conclusion, ACE I/D polymorphism is associated with an increase in the case numbers and unfavorable clinical outcomes in patients with the SARS-CoV-2 infection. Genotype DI of ACE insertion/deletion polymorphism is associated with the infection rate of severe pneumonia in this study, conducted in the Turkish population. Therefore, the presence of the D allele in both homo/heterozygous forms could contribute to COVID-19 severity. Hopefully, future experimental and clinical studies shed light on the importance of the genomics of RAS molecules for better management of patients with COVID-19.

## Figures and Tables

**Table 1:** List of primer sets.

Primers	Sequences (5′ to 3′)
ACE F (1^st^ reaction)	CTGGAGACACTCCCATCCTTTCT
ACE R (1^st^ reaction)	GATGTGGCCATCACATTCGTCAGAT
Insertion specific ACE F (2^nd^ reaction)	TGGGACCACAGCGCCCGCCACTAC
Insertion specific ACE R (2^nd^ reaction)	TCGCCAGCCCTCCCATGCCCATAA

**Table 2:** The distribution of the genotypes in COVID-19 and control groups.

Genotype	COVID-19 group	Control group^∗^	*χ* ^2^	*p* value
DD	45 (40%)	77 (26%)	46.6	<0.00001
DI	59 (53%)	95 (32%)		
II	8 (7%)	128 (42%)		
*Allele*				
D	149 (67%)	249 (42%)	40.9	<0.00001
I	75 (33%)	351 (58%)		

Control frequencies were obtained from literature on Turkish population [13].

**Table 3:** Comparison of the clinical characteristics and comorbidities of the COVID-19 patients according to alleles.

	Alleles			*p*
	DD	DI	II	
Median age (years)	48.2 [19-83]	47 [19-84]	40.5 [30-50]	0.45
Gender *n* (%)				
Female	28 (62.2)	32 (54.2)	6 (75)	0.45
Male	17 (37.8)	27 (45.8)	2 (25)	
Comorbidity *n* (%)				
Hypertension	10 (22.2)	10 (16.9)	—	0.38
Diabetes mellitus	5 (11.1)	7 (11.9)	1 (12.5)	0.99
Coronary artery disease	4 (8.9)	2 (3.4)	—	0.36
Dyslipidemia	2 (4.4)	3 (5.1)	—	0.80
Hypotyroidism	2 (4.4)	2 (3.4)	—	0.81
Malignancy	2 (4.4)	2 (3.4)	—	0.81

Brackets indicate minimum and maximum ranges of ages. *n*: number of patients; %: percent.

**Table 4:** Comparison of the clinical and laboratory characteristics of the COVID-19 patients at admission according to alleles.

	Alleles			*p*
	DD	DI	II	
*Pneumonian(%)*				0.021
Severe	2 (8)	10 (31.3)	—	
Nonsevere	23 (92)	22 (68.7)	8 (100)	
Need for oxygen *n* (%)	3 (6.7)	12 (20.3)	—	0.06
Presence of fever *n* (%)	23 (51.1)	27 (45.8)	3 (37.5)	0.73
Median body temperature (°C)	38 [36.5-39.7]	37.7 [36.4-39.9]	37.8 [36.6-39.1]	0.92
Ferritin (*μ*g/L)	54.2 [5.3-3704]	49.9 [2.7-1300]	64.5 [9.1-273]	0.93
D-dimer (mg/L) [median]	0.36 [0.2-2.8]	0.38 [0.2-1.9]	0.36 [0.2-0.7]	0.91
CRP (mg/dL)	0.76 [0.1-24.6]	0.81 [0.1-14]	0.76 [0.12-1.35]	0.97
NLR	2.6 [0.89-14.5]	2.7 [0.66-14.1]	1.9 [0.83-4.34]	0.47
LDH (U/L)	165 [120-477]	183 [122-449]	188 [110-272]	0.59
CK (U/L)	76.5 [9-311]	83 [16-1648]	69.5 [35-150]	0.34
AST (U/L)	22.5 [15-98]	25 [12-81]	29 [17-56]	0.51
Total bilirubin (mg/dL)	0.45 [0.24-0.98]	0.42 [0.1-1.39]	0.44 [0.15-0.90]	0.76
BUN (mg/dL)	13.3 [5.7-43.5]	12.2 [4.1-28.3]	8.84 [7-18.1]	0.1
Creatinine (mg/dL)	0.7 [0.49-3.65]	0.79 [0.44-1.2]	0.67 [0.55-0.96]	0.4
ESR (mm/h)	13 [2-62]	12 [2.7-56]	15 [2-27]	0.89

AST: aspartate aminotransferase; BUN: blood urea nitrogen; CK: creatinine kinase; CRP: C-reactive protein; ESR: erytrocyte sedimentation rate; LDH: lactate dehydrogenase; NLR: neutrophile to lymphocyte ratio. *μ*g/L: micrograms per liter; mg/L: milligrams per liter; U/L: units per liter; mg/dL: milligrams per decilitre; mm/h: millimeters per hour. Brackets indicate minimum and maximum ranges. Parentheses indicate percent value.

**Table 5:** Comparison of the main outcomes in the COVID-19 patients according to alleles.

	Alleles			*p*
	DD	DI	II	
Time to defervescence (days)	3 [1-27]	3 [1-29]	3 [5-10]	0.09
Duration of hospitalisation (days)	9 [4-77]	8 [1-46]	10 [5-10]	0.91
Mortality	1 (2.2%)	4 (6.8%)	—	0.43

Brackets indicate minimum and maximum ranges.

## Data Availability

All data generated or analyzed during this study are included in this published article.
